# Supplementary material for: Metabolic Alterations in Human Post‐Mortem Frontal Cortex and Cerebrospinal Fluid Associated With High Levels of Nicotine Metabolite Cotinine
Source: Addict Biol. 2025 Jun 20;30(6):e70064. doi: 10.1111/adb.70064 (PMC12179806; doi:10.1111/adb.70064)
Supplement: Supplementary file 1 — Figure S1. Correlations of metabolites with post‐mortem interval (PMI). Pearson’s correlation analysis figures of FC (row A) and CSF (row B) with PMI. [file ADB-30-e70064-s001.pdf]

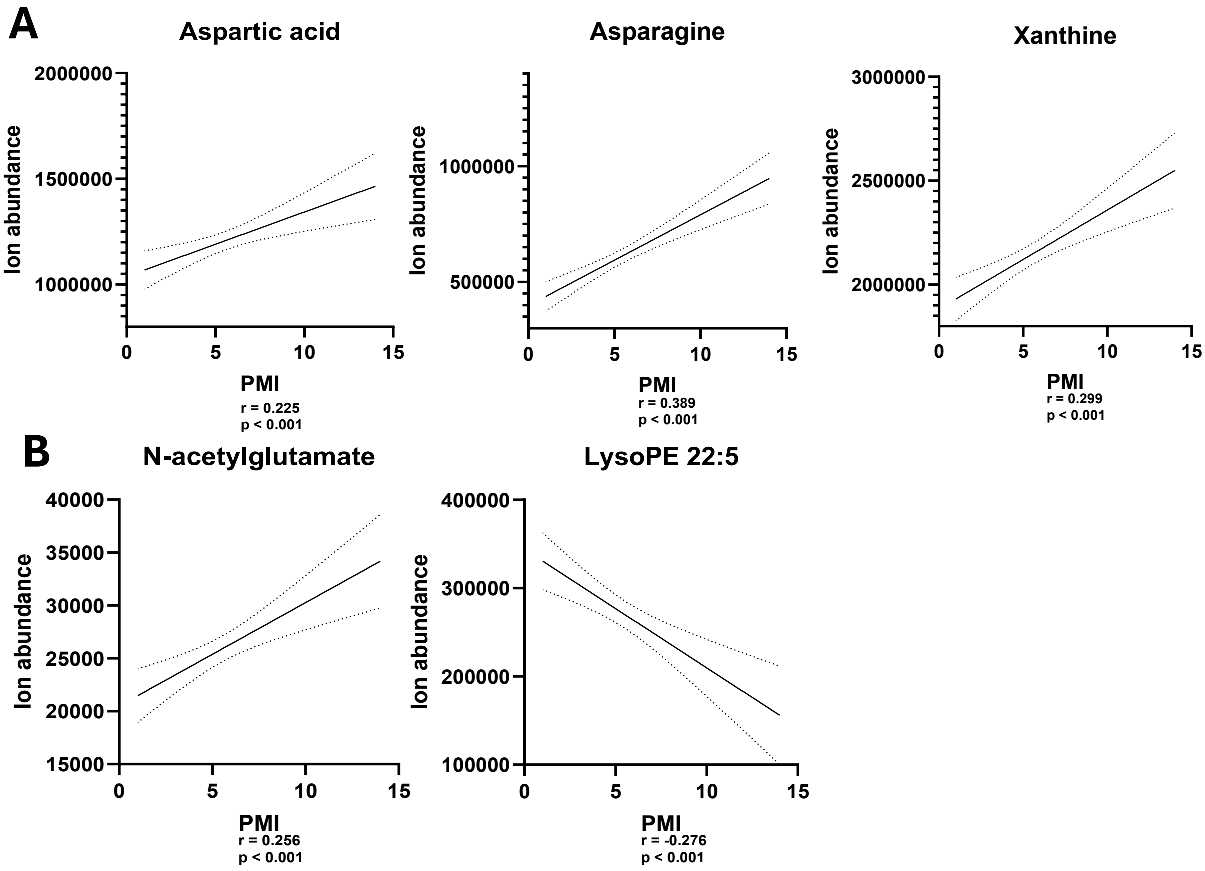

Supplementary Figure 1: Correlations of metabolites with post-mortem interval (PMI). Pearson's correlation analysis figures of FC (row A) and CSF (row B) with PMI.
